# Developing an Online Tool to Measure Social Network Structure and Perceived Social Support Amongst Autistic Students in Higher Education: A Feasibility Study

**DOI:** 10.1007/s10803-019-04070-5

**Published:** 2019-05-22

**Authors:** Jiedi Lei, Chris Ashwin, Mark Brosnan, Ailsa Russell

**Affiliations:** Centre for Applied Autism Research, Department of Psychology, University of Bath, Claverton Down, BA2 7AY Bath, UK

**Keywords:** Autism spectrum disorder, Social network, Perceived social support, University, College, Transition

## Abstract

The academic, daily-living, and social challenges all students face during university transition can become magnified for many autistic students, who might struggle to adapt to changes in their social network structure (SNS) and perceived social support (PSS). This study assessed the development, feasibility, and convergent validity of a novel online tool (Social Network and Perceived Social Support—SNaPSS) designed to quantitatively and qualitatively evaluate SNS and PSS during university transition. SNaPSS demonstrated good feasibility for completion amongst autistic students (Study 1, n = 10, 17–19 years), and adequate convergent validity against other PSS, autism symptom severity, and social anxiety measures amongst autistic (n = 28) and typically developing students (Study 2, n = 112, 17–19 years). Broader implications of SNaPSS to measure SNS/PSS are discussed.

## Introduction

“No man is an island, entire of itself; every man is a piece of the continent, a part of the main…” (Donne 1839, p. 574). Indeed, for any individual, social networks function to provide practical and social support, both of which are critical for maintaining wellbeing (Dunbar and Spoors 1995; Hill and Dunbar 2003; Roberts and Dunbar 2011; Siedlecki et al. 2014). Significant transition points in an individual’s life such as moving from one part of the education system to another, changing jobs, geographical location etc. are accompanied by an inevitable change in one’s social network and access to social support. Developing a new social network can be critical to successful transition and adaptation to a new set of life circumstances.

An important transition many young people experience is the move to post-secondary education (such as university or college), which often coincides with the first time of leaving home and leading an independent life (Fisher and Hood 1987). Previous studies have found that for students transitioning to university, better perceived social support (PSS) was associated not only with better adjustment outcomes, but also better mental and physical health, greater life satisfaction and more positive coping (Gall et al. 2000; Tao et al. 2000). Students also experience changes in the kinds of support they receive from different social network members. For example, high quality support from family (Hays and Oxley 1986) was associated with better adjustment at university (Friedlander et al. 2007), though support was more in the form of fun/relaxation, rather than informational/emotional support (Swenson et al. 2008). In contrast, peers provided more social and emotional support to students during transition (Friedlander et al. 2007), which helped to improve students’ mental health, and encouraged students to use more positive coping strategies (Tao et al. 2000). Therefore, social network structure (e.g., who is in the social network) and perceived social support are crucial for enabling successful transition to university for students. The focus of the present study is to identify the feasibility of assessing these variables for autistic^1^ students transitioning to university. Furthering our understanding of factors critical to successful social network transition can shape the provision of supportive interventions for stakeholders in education.

### Social Network Analysis

Social network analysis (SNA) is the quantitative evaluation of both structural and functional components of the types of relationships an individual has with other people around him/her (Kreider et al. 2016; Scott 2017). Some important structural social network components are size (i.e., how many people an individual may be in contact with); composition (i.e., the types of relationships an individual has with each member, such as family, friends, etc.); density (i.e., the extent to which individuals named within a network might know each other); and centrality (i.e., the location of an individual within his/her social network). Functional components of social network comprise the extent to which an individual might receive, or perceive support from different social network members. There are some correlations between structural and functional components of social networks. For example, during a stable phase of one’s lifetime, having a high-density social network might increase the accessibility of information and resources through improved flow through different social network members. However, during a major life transition such as starting university or moving across countries, having a low-density social network might increase one’s resilience to adapt to changes in environment, as the individual may be able to maintain some existing social contacts rather than lose access to the entire social network (Scott 2017).

There are two main types of SNA, sociomap and ecomap. Sociomap (Correa and Ma 2011) is usually measured within a pre-defined social space where it is assumed that all individuals have the potential to interact with each other and establish relationships (e.g., within a school classroom). All individuals are sampled and a summary combining all the reported relationship information is used to generate a sociomap. In contrast, an ecomap (Ray and Street 2005) focuses on a particular individual, and assesses the relationships that this individual considers to be important to him/her in their personal environment. Therefore, compared to sociomap, ecomap produces a much more individualised visualisation of one’s personal social network across multiple domains (e.g., friends, family, work colleagues etc.), though may be more subject to self-reporting bias. A particular strength of ecomap is that it can capture changes in one’s social network during life transitions, when the sudden change in environment no longer provides a clearly defined space for sampling information to generate a sociomap.

### Social Network Analysis in Post-secondary Education

The ability to establish a novel social network, especially with same-aged peers, become increasingly important over the course of development (Kamps et al. 1997). Adolescents begin to rely more on friends and less on family for a wide variety of support including both informational/practical as well as personal/emotional support (Lee and Goldstein 2016). This shift to increasing independence from family members is not always gradual and ‘sudden’ events such as a move out of home to access post-secondary level education accelerates the process. In such situations, higher levels of perceived social support is often associated with better transition outcomes in typically developing students (Azmitia et al. 2013; Friedlander et al. 2007).

There have been few studies using measures that simultaneously capture both structural and functional dimensions of social networks specifically amongst students transitioning to university. One recent systematic review of the literature (Lei et al. in preparation) which followed PRISMA guidelines (Moher et al. 2009) assessed how changes in both SNS and PSS during transition to first year of university might be associated with transition outcomes in students aged 17–21, using longitudinal research design. After title, abstract, and full text screening, as well as quality appraisal, the review identified a total of only ten studies that assessed either changes in SNS or PSS (or both) amongst first year university students. Only one of the ten studies was found that simultaneously measured both changes in SNS and PSS in first year university students (Hays and Oxley 1986). Hays and Oxley (1986) asked participants to report up to ten people that they had seen in the past 3 weeks, and then reported whether each individual had provided emotional, tangible, and fun support.

Although the authors were able to capture some structural and functional aspects of students’ social networks, the types of support were more general (e.g., “providing comfort/support during a personal issue”), rather than specifically focusing on the challenges faced by students transitioning to university. The functional support measured also did not take into account differences in perceived quantity and quality of each type of support provided, making it difficult to identify whether individual differences in transition outcomes might be associated with perceived quantity and/or quality of support in any specific area. Therefore, developing a tool that can not only capture both the structural and functional aspects of students’ social networks, but also capture support domains most relevant to challenges faced by students transitioning to university, and differentiate between perceived quantity and quality of support can enable university stakeholders to better understand *who* is best at providing *which* types of support during the transition process.

### Autism, and Social Network Analysis

Autism spectrum disorder (ASD, hereafter autism) is a neurodevelopmental disorder characterized by differences in social communication and a pattern of restrictive and repetitive behavior, interests and activities (American Psychiatric Association 2013). Autism affects one in 59 children (CDC 2018). Autism affects an individual across the lifespan and research findings suggest that although many autistic people report a desire for social relationships, they reported reduced numbers of social relationships and more adverse social events such as peer victimization than other groups (Jackson et al. 2018a). During early and middle-childhood, a structured educational system and parental support can be influential in scaffolding the development of social networks (Kreider et al. 2016). However, difficulties associated with social transition for many autistic students emerge from a young age, as a recent systematic review (Nuske et al. 2019) that examined 27 studies on school transitions for 443 autistic students found that students experienced high levels of anxiety, as well as greater mental health needs, sensory, behavioral, and academic challenges when transitioning to a new school. Autistic students reported greater social pressure post-transition, and found forming new friendships especially anxiety provoking in light of their social communication difficulties, and some reported experiences of bullying and isolation (Nuske et al. 2019).

In previous research analyzing social networks of both autistic and typically developing (TD) children in mainstream classrooms (Anderson et al. 2016; Chamberlain et al. 2007; Locke, et al. 2010; Locke et al. 2013; Rotheram-Fuller et al. 2010), researchers have frequently used the “Friendship Survey”—which asks each child in a classroom to freely recall names of children who like or dislike to hang out with each other. The information collected can be used to generate a sociomap consisting of friendship clusters within the class, and be used to measure network centrality of any specific child within the classroom to reveal the extent of inclusion. Although this method can successfully capture the social networks within a single classroom setting it can not adequately capture the inherent changes in social network as students make the transition to university where socialization becomes more complex, with less focus on classroom based interaction and greater diversity of extra-curricular social forums, Therefore, it may not be feasible to gather information from *all* social network members to generate accurate sociomaps to reflect changes in social network across time at university, but an ecomap may be more appropriate.

### Autism and Post-secondary Education

Transition into adolescence and early adulthood can present challenges for all young people, and might be especially challenging for autistic students to navigate. Although almost half (46%) of autistic individuals have average or above average IQ (CDC 2018), and have the intellectual potential to attend postsecondary education (Sanford et al. 2011), attendance in post-secondary education amongst autistic students is relatively poor. In the U.S., it is estimated that only around 35% of autistic students can complete their post-secondary education, which is lower than the 38% of graduation rate for students with other disabilities, and 51% of typically developing peers (Gobbo and Shmulsky 2014). Similarly in the UK, fewer autistic students graduated from university with 2:1 or first class honors degree (62.8%) compared to student with other forms of disabilities (66%), and typically developing peers (68.1%) (Lucas and James 2018).

Although the number of autistic students enrolling in postsecondary education have increased in recent years, mental wellbeing amongst students on campus is relatively poor (Jackson et al. 2018a, b), with between 47 and 71% of autistic students experiencing high levels of anxiety, loneliness, and symptoms of depression (Gelbar et al. 2014). In particular, participation in social activities, especially with same-aged peers, can often be especially poor amongst autistic young adults compared to young people with other forms of special education needs (Orsmond et al. 2013). Reduced participation in social activities was particularly evident amongst autistic young people with poorer conversation skills and functional ability, and the absence or poor quality friendships can lead to greater feelings of social isolation amongst autistic students (Orsmond et al. 2013).

This high occurrence of loneliness suggests that the ability to successfully establish a new social network at university and seek out appropriate sources of social support can be especially challenging for autistic young people (Adreon and Durocher 2007), and highlights that better quality and more tailored support to meet individuals’ needs for this vulnerable student population at university is much needed, especially to monitor students’ interactions with same-aged peers, which may help to buffer against feelings of loneliness and isolation. Understanding the changes in social network structure (SNS) and perceived social support (PSS) during transition to university might offer insight into students’ ability to successfully adapt to the novel environment, given that high levels of perceived social support is often associated with better transition outcomes (Azmitia et al. 2013; Friedlander et al. 2007).

For many autistic students at university, family members (especially parents) often continue to provide high levels of support across a range of academic, daily living, and socialization areas (Elias et al. 2017; Fleischer 2012; Mitchell and Beresford 2014). Continued high levels of support from family might therefore compensate for potentially lower levels of perceived social support from same-aged peers at university, reflecting differences in both SNS and PSS between autistic students and their TD peers at university. To date, no studies have yet explicitly evaluated autistic students’ perception of their PSS from different social network members. Gaining a better understanding of both the structural and functional social support network of autistic students can help stakeholders adopt a more systemic approach when planning transition supports for autistic students, and to better integrate different resources such as family, peers, and university staff to optimize the support structure for autistic students at university.

## Development of Social Network and Perceived Social Support (SNaPSS) tool

The SNaPSS is developed to capture both key structural components of one’s social network (size, composition, and density) (Scott 2017), as well as the functional PSS provided by each network member within one’s social network. The rationale behind the SNaPSS is to develop an easy-to-use online tool that can help students visualize a holistic view of their perceived social world, as well as help relevant stakeholders to effectively gather information about the quantity and quality of social relationships that each student perceives to be the most important to them. The SNaPSS is based on ecomap methods, and was developed to evaluate structural and functional aspects of social network i.e. Social Network Size (SNS) and Perceived Social Support (PSS) for students making the transition to university. SNaPSS aims to capture: (1) a wide range of social network structures consisting of network members that students consider to be important to them; (2) perceived frequency and quality of social support provided by all social network members across a wide range of academic, daily living, and socialization areas related to challenges that students might face during transition to university.

### SNS Measure Development

In order to adopt an ecomap approach to evaluate SNS and PSS, the SNaPSS first gathered information about each network member (alter) within an individual’s (ego) social network, such as basic demographic information (e.g., name, sex, relationship to ego), as well as the relationship between each network member with other network members (alter–alter relationship). The former gives an approximation of the individual’s social network size and network composition, whilst the latter provides a measure of network density. Next, individuals reported the types, frequency, and quality of support they perceived to have been provided by each network member named, which provides various measures of PSS that can be broken down either by types of support received (e.g., academic, daily living, and socialization), or by types of network members who have provided the support (e.g., family, friends, and other network members).

Given that for each network member named, there is a wealth of information collected based on that individual (demographics, alter–alter relationships, PSS), the length of the questionnaire using an ecomap approach can quickly accumulate and become too long and not feasible for students to complete. Therefore, a balance needs to be struck between the number of network members that students can include within their social network, and the number of questions answered per network member.

Prior research have suggested that although human networks can range from 130 to 250 individuals (Hill and Dunbar 2003), the closer and more intimate inner circle which provides most functional support and who are in regular contact to individuals stands at around 10–15 individuals (Dunbar and Spoors 1995). Given that SNS is also a dynamic construct that can change over time, measurements of SNS also need to define a specific period of time for participants to recall their SNS. In a previous study that evaluated SNS in college students in the US, Hays and Oxley (1986) asked students to report up to ten network members that they considered to be close to them and have been in contact with for the past 3 weeks. However, the two potential limitations are that (1) ten network members is smaller than the upper limit of intimate social circles found by prior research (Dunbar and Spoors 1995), thus potentially limiting the ability of students with larger social networks to accurately report their inner social circle; (2) 3 weeks is a relatively short time window to measure the establishment of new social network ties during a major life transition such as going to university, especially when used in longitudinal designs to reflect changes in SNS over time.

The current SNaPSS overcomes these limitations by asking each participant to name up to 20 individuals with whom they have been in contact with over the past 3 months, and whose relationships were considered to be particularly important and worthwhile to the participant, giving an approximation of social network size. The choice of 20 network members is greater than the average upper limit of number of network members included in the intimate circle (Dunbar and Spoors 1995) to try to minimize ceiling effects. The duration of 3 months was chosen as it provides a significant time frame for students to establish and develop new social ties, and approximately corresponds to the duration of an academic term at university. The use of academic term as a time frame for recalling changes in SNS is particularly important for longitudinal studies that might investigate changes in SNS across first year university transition. A longer time frame can help capture significant changes in SNS that might occur over an academic term, as students might participate in different social events, clubs and societies throughout the academic year.

### PSS Measure Development

Preliminary items focusing on PSS for the SNaPSS were developed based on prior literature to capture areas where students in transition might require support, as well as areas that might be especially challenging for autistic students. Autistic students attending university often face challenges in a wide range of social, daily living, and academic areas (Adreon and Durocher 2007). For socialization, autistic students often experience difficulties in perspective taking and gauging the interest of their audience when communicating with others (Baron-Cohen 1989; Baron-Cohen et al. 1985; Zager and Alpern 2010), and can often miss out on or misinterpret nonverbal social cues during a social interaction. Communication with purely social intent can also be lacking in autistic people e.g. engaging in ‘small talk’ as a tool for social reciprocity. In addition, some autistic people have restricted and circumscribed interests that can limit their ability to engage in conversations across a varied range of topics that may lie outside of their interests, and can further interfere with their social interactions with other people. Such social communication deficits can reduce autistic students’ ability to socialize with peers across a variety of contexts, ranging from living in shared accommodation, to completing coursework that requires working in groups (Hees et al. 2015).

Many autistic students also experience difficulties in many executive functioning (EF) processes such as planning and organization (Ozonoff et al. 1991). In a recent meta-analysis that assessed the extent of impairments across different EF subdomains in autistic people, Demetriou et al. (2018) found a moderate effect size for impairments across all EF subdomains, highlighting the nature of global EF deficits observed across development in autistic people. EF deficits can further impair one’s ability to live independently, given that many daily tasks requires one to seek out relevant information, synthesize a plan, and follow through the plan in a series of steps in order to achieve the final goal (Gilotty et al. 2002). EF deficits can therefore affect a wide range of daily living skills such as managing one’s finances, cooking, and doing laundry, as well as academic demands such as meeting coursework deadlines and managing one’s time (Hewitt 2011; Pugliese et al. 2015; Rosenthal et al. 2013; Sparrow et al. 2005).

Based on prior literature, a list of 15 preliminary areas of support (five academic, five daily living, and five socialization) were developed (Table 1), and piloted during part one of the current study to assess face validity of these items in relation to concerns and worries that autistic students have when transitioning to university.

**Table 1:** Areas of support across academic, daily living, and socialization domains that are included in social network and perceived social support (SNaPSS) measure

Academic	Daily living	Socialization
1. Course workload	1. Changes in my routine	1. Living in shared accommodation
2. Course difficulty	2. Cooking	2. Getting on with people I live with
3. Meeting course deadlines	3. House chores (laundry, cleaning/tidying/organising room)	3. Fitting in
4. Doing group work	4. Manage/budget my finances	4. Being bullied/feeling isolated
5. Time management and routine	5. Self-care/seeking medical advice	5. Socializing with other students/making friends

## The Current Study

The current study develops and assesses the feasibility of a novel online tool, social network and perceived social support (SNaPSS). This study is conducted in two parts. The first part of the study considered issues of feasibility of administration, ease of completion and measurement of individual differences in both SNS and PSS in a small group of autistic students. The second part of the study used a larger sample of TD and autistic students who were about to transition to university, and evaluated convergent validity between the SNaPSS and other measures of PSS, anxiety, and levels of autistic-like traits.

Therefore, the overall aim of the study is to develop, test the feasibility, and assess convergent validity of a novel online tool designed to measure both SNS and PSS amongst autistic and TD students transitioning to higher education. The research questions examined were as follows for “Part 1” and “Part 2” sections of the study:

1. Is the SNaPSS a feasible tool for autistic students to complete online? (Part 1).
2. Can the SNaPSS effectively capture individual differences in SNS and PSS? (Part 1).
3. Does the SNaPSS show convergent validity with current measures of PSS, autistic traits, and social anxiety? (Part 2).

## Methodology

Both parts of the study were approved by the University’s departmental ethics committee and is in line with the Declaration of Helsinki as revised in 2000. All participants provided written informed consent prior to participating in the research study.

### Part 1

#### Participants

For part one of the study assessing feasibility of SNaPSS and face validity of preliminary items, participants included ten students (two female, eight male) between the ages of 17–19 years old who took part in an Autism Summer School programme that supported autistic students to transition to university (Table 2). All participants who enrolled at the Autism Summer School had received a prior diagnosis of Autism, Asperger’s, or Autism Spectrum Disorder from a trained clinical professional. Prior to arriving at the Autism Summer School, parents also completed the Social Communication Questionnaire (SCQ; Rutter et al. 2003) to further inform diagnosis and autism symptom severity.

**Table 2:** Study Part 1: participant demographics for feasibility study (N = 10)

	Mean (SD)	Range
Age (years)	17.90 (0.74)	17–19
Social communication (SCQ total)^a^	21.20 (5.87)	14–33
Social network size^b^	11.20 (6.49)	5–20
Family (n)	3.80 (1.93)	2–7
Friends (n)	5.90 (5.09)	0–13
Other (n)	1.50 (1.78)	0–4
Social network density^c^	0.55 (0.28)	0.88–0.05
Perceived distress frequency^d^		
Academic	4.20 (2.55)	0.2–9.20
Daily living	2.5 (2.12)	0.4–6.60
Social	3.31 (3.16)	0.2–11.40
Perceived overall support availability^e^		
Academic	6.78 (3.05)	1–12
Daily living	5.51 (2.85)	2–12
Social	5.22 (2.04)	2–8.05
Perceived overall support quality^f^		
Academic	5.49 (2.57)	1–9
Daily living	4.34 (2.38)	1–9
Social	3.95 (1.95)	1–7.80
Support frequency from social network^g^		
Family	6.59 (3.02)	2–11
Friends	2.04 (2.03)	0–5.25
Other	1.84 (2.65)	0–6.67
Support quality from social network^h^		
Family	8.70 (3.98)	3–14
Friends	3.73 (3.70)	0–9.5
Other	3.00 (4.38)	0–11.33

*SCQ* Social communication questionnaire

^a^SCQ is scored between 0 and 39, cut-off is 15

^b^Social network size is scored between 0 and 20

^c^Social network density is scored between 0 and 1

^d^Perceived distress frequency is scored between 0 and 20

^e^Perceived overall support availability is scored between 0 and 20

^f^Perceived overall support quality is scored between 0 and 20

^g^Support frequency is scored between 0 and 15

^h^Support Quality is scored between 0 and 15

#### Measures

##### Social Network and Perceived Social Support Tool (SNaPSS)

The SNaPSS is a novel online self-report tool developed by the first author to assess both social network structure (SNS) and perceived social support (PSS) amongst students transitioning to university. The tool is divided into three sections. First, participants reported perceived frequency of distress (stress, anxiety, and depressed/low mood) across a total of 15 academic, daily living, and socialization areas (Table 1). Participants rated frequency of distress (stress, anxiety, feelings of low mood) on a 5-point scale ranging from 0 (never) to 4 (6 or more times a week). For each area endorsed as being associated with distress, participants rated both whether they perceived there were people they could turn to for support (i.e., support availability), as well as how supported they felt (i.e., support quality). Participants rated both support availability and support quality on a 5-point scale ranging from 0 (never) to 4 (always).

Second, for SNS, each participant named up to 20 individuals with whom they have been in contact with over the past 3 months, and whose relationships were considered to be particularly important and worthwhile to the participant, giving an approximation of social network size. Participants then reported the type of relationship (e.g., family, friends, other individuals such as teacher/lecturer, support/social worker etc.), the degree of similarity, the frequency, and modes of contact between self and each individual named. Participants also reported whether to the best of their knowledge, any two individuals named knew of each other, giving an indication of the social network density, scored between 0 (low) and 1 (high), with high density reflecting most individuals within a social network know of each other. Size and density of social networks can also be represented visually using a social network map (ecomap).

Finally, for PSS, participants reported the types of support provided by each social network member across the academic, daily living, and socialization areas (Table 1) over the past 3 months. Of the types of support endorsed by each social network member, participants then reported: (1) the frequency of perceived support on a 5-point scale (1 = once/twice in total; 5 = 6 or more times/week); (2) the quality of perceived support on a 5-point Likert scale (1 = not at all supported; 5 = very much supported). For each category of social network members (i.e., family, friends, and others—including teachers/lecturers/tutors, social/support worker, other), perceived frequency and quality of support was calculated as the average value of those members who endorsed at least one type of academic, daily living, or socialization support. Network members who did not provide any types of support were scored as 0 for both perceived frequency and quality of support. Therefore, total perceived frequency and quality of support are scored between 0 and 15, with 0–5 within each of academic, daily living, and socialization domain.

##### Social Communication Questionnaire—Lifetime (SCQ, Rutter et al. 2003)

The SCQ Lifetime is a parent-report 40-item questionnaire that assesses whether the individual has displayed symptoms associated with ASD, such as social communication difficulties, throughout their lifetime. Each item is scored using a dichotomous 0 (never been present) to 1 (have been present) scale. A total score above 15 indicates that the individual is likely to have Autism Spectrum Disorder (sensitivity = .68, specificity = .41) (Hanson et al. 2002), and may require further testing to assess diagnosis. The SCQ has good internal consistency (Cronbach’s alpha = .67–.90 for the subscales and total score), and has good convergent validity (Pearson’s correlation = .55–.59 for the subscales) with the Autism Diagnostic Interview—Revised (a gold standard autism diagnostic tool) (Berument et al. 1999).

##### Study Design

For part one of the study, all students participating in the Autism Summer School were invited to take part in the current study. The Autism Summer School is a programme held at the [anonymized for review] aimed to inform autistic students about university life, and support university transition (Lei et al. 2018). Parents of all participants completed the SCQ as part of the pre-summer school arrival questionnaire pack. On the first day of the summer school during 2017, all students were invited by the first author to take part in the pilot study, after having received a presentation from the first author on social changes during transition to university. The aims of the pilot study were clearly explained to students prior to participation, including that their participation was voluntary, and their decision to take part in the study (or not) will not affect their participation in the summer school programme. A total of eleven students volunteered to enroll in the study. Written informed consent was obtained from all students prior to participation. One student failed to complete the session and withdrew from the study due to experiencing high levels of social anxiety during his time at the Autism Summer School. The remaining ten students completed the novel online tool via Qualtrics, and were asked for verbal and written feedback on their thoughts about the format, language, and appropriateness of the support areas included in the tool as a measure of SNS and PSS after they have completed the SNaPSS.

### Part 2

#### Participants

For Part 2 of the study assessing convergent validity of SNaPSS with other measures of PSS, social anxiety, and autistic traits, a larger sample of 112 TD students (20 M, 92 F) and 28 autistic students (14 M, 14 F) who were about to transition to university were recruited separately as part of a longitudinal study that assesses changes in SNS and PSS during first year of university. Convergent validity is assessed between the measures taken at baseline (i.e., during the first 2 weeks of first term at first-year of university), including Multidimensional Scale for Perceived Social Support (Zimet et al. 1988), Social Anxiety Scale for Adolescents (La Greca et al. 2015), and Autism Quotient-28 (Hoekstra et al. 2011).

#### Measures

##### Social Network and Perceived Social Support Tool (SNaPSS)

The SNaPSS (as described in “Part 1” section) used for “Part 2” section of the study was a slightly revised version based on students’ feedback from Part 1 feasibility study. The adaptations are described in greater length in the results from feasibility study, and also in discussion. First, a question about whether or not students have taken a gap year^2^ was added. Second, feelings of stress, anxiety, and low mood were combined into a single “distress” rating using the same scoring system.

##### Multidimensional Scale for Perceived Social Support (MSPSS, Zimet et al. 1988)

The MSPSS is a 12-item self-report measure of perceived social support from family, friends, and significant other. The items are listed as statements surrounding more general provision of support and especially emotional support. Example statements include “I get the emotional help and support I need from my family” (Family), “I have friends with whom I can share my joys and sorrows.” (Friends). Each item is rated on a 7-point Likert scale (1 = very strongly disagree, 7 = very strongly agree). The MSPSS has good internal consistency (Cronbach’s alpha = .85–.97 for the subscales and total score).

##### Autism Quotient-28 (AQ-28, Hoekstra et al. 2011)

AQ-28 is an abridged version of the full 50-item Autism Quotient scale, with a list of statements which refer to social behaviors related to autistic traits, such as “I prefer to do things the same way over and over again”, with each item rated on a four-point Likert scale (1 = definitely agree, 4 = definitely disagree). The abridged scale has good internal consistency (Cronbach’s alpha = .77–.86), and high predictive validity, with scores > 65 having a sensitivity of .97 and specificity of .82.

##### Social Anxiety Scale for Adolescents (SAS-A, La Greca et al. 2015)

SAS-A is a 22 item self-report measure of social anxiety in adolescents, with three subscales that assess (1) fear of negative evaluation (FNE; eight items); (2) social avoidance and distress in social situations (SAD-NEW; six items); and (3) generalized social avoidance and distress (SAD-G; four items). SAS-A has high internal consistency (Cronbach’s alpha .77–.92), good concurrent validity with measures of social phobia, good discriminant validity, and good test–retest reliability.

##### Study Design

For the second part of the study, first year university students were recruited for a larger longitudinal study examining changes in social networks via advertisements on campus such as posters, social media, and information given at lectures at a medium sized university in the UK during the first 2 weeks of the university term in September/October. Typically developing (TD) and autistic students in their first term at University were recruited. Autistic students must have a diagnosis of ASD by a clinical professional, and TD students are defined as not having any other concurrent mental, physical, or other health conditions at the time of enrolment. Participants were provided with study information via Qualtrics, and then completed consent forms and all questionnaires online via Qualtrics within the first 2 weeks of university, as part of the baseline data collection for the longitudinal study. A total of 112 TD students and 28 autistic students were recruited to take part in the larger longitudinal study, which asked students to complete a collection of questionnaires assessing how changes in students’ SNS/PSS (as assessed by SNaPSS) might influence their university transition outcomes. The larger longitudinal study asked students to complete questionnaires during September (time point 1), December (time point 2), and March (time point 3) of first year of university. For Part 2 of the current study, only the cross-sectional data collected during time point 1 (September of first year of university) were used for the purpose of assessing convergent validity. Data collection for the longitudinal study was still ongoing and incomplete at time of submission for the current study.

### Data Analyses

All data analyses were completed using SPSS version 24 (IBM SPSS Statistics 2016), and Gephi2 (Bastian et al. 2009) was used to calculate both social network density, and for generating individual ecomaps for social network structure.

For Part 1 of the feasibility study, we first assessed participants’ verbal and written feedback on feasibility of the online tool, and any suggestions they had on the language, format, and delivery of SNaPSS. Where appropriate, adaptations were made to SNaPSS based on participants’ feedback. Second, we explored the range of scores reported by participants on the frequency of distress across academic, daily living, and socialization areas, as well as in perceived availability and quality of support during times of distress across each area. We used the non-parametric Friedman test as an alternative to one-way repeated measures ANOVA, due to the small sample size (N = 10) in the current study, and Wilcoxon sign ranked test for post hoc analyses. We used Bonferroni to correct for multiple comparisons. Third, using Gephi2, we explored whether the questions on social network structure can elicit participants to disclose a wide range of SNS size and density. We then evaluated whether participants’ social communication impairments would be associated with their social network structure, and we conducted a Pearson’s correlation between participants’ SCQ total score, SNS size and density, as well as social network composition. Finally, we assessed whether differences emerged in frequency and quality of support provided by family, friends, and other social network members by conducting the non-parametric Friedman test. We used Bonferroni to correct for multiple comparisons.

For Part 2 of the study, we assessed concurrent validity by conducting Pearson’s correlation between SNaPSS’ PSS scores for family and friends with the family and friends subscale of the MSPSS in both TD and autistic students. To assess the relationship between SNS and social competency factors such as level of social anxiety and autistic traits, we also conducted Pearson’s correlation between social network size, density, composition, and SAS-A and AQ-28 total scores.

## Results Part 1: Assessing Feasibility of SNaPSS

### Feasibility and Use of Language

Participant demographics for Part 1 of the study can be found in Table 2. With the exception of one participant who withdrew from the study due to experiencing high levels of social anxiety during the Autism Summer School, the remaining ten participants all successfully completed the online tool within 12–47 min (M = 28.22; SD = 9.20). Overall, participants found the questionnaire to be clear in its format and use of language, and the questions were relevant and appropriate. Participants’ feedback on how to improve the questionnaire included three main areas. First, four participants commented on combining the questions on stress, anxiety, and depressed/low mood into a single question that asked about general perceived distress related to each area. Participants felt that the distinction between stress and anxiety in particular was not very clear, and that by combining the questions into a single distress question can both shorten the duration of the questionnaire and make it less repetitive. Second, two participants commented that having to name at least five individuals and up to 20 maximum might be potentially too many for some people, and that a period of 3 months is a long timeframe for them to recall social interactions. However, students commented that the poor recall might be due to the nature of having been on summer holidays for the past 3 months at the time of questionnaire completion, and it might be easier to recall social interactions during more structured term time. Finally, one participant commented on having an option about having taken a gap year, as she did not require any academic support in the past year due to not being in full time education.

### Perceived Distress Across Academic, Daily Living, and Socialization

A range of perceived distress (stress, anxiety, and depressed/low mood) across academic, daily living, and socialization areas varied largely amongst participants, as well as the perceived availability and quality of support for those who endorsed distress are reported in Table 2. Using Friedman’s test, no significant differences were observed for perceived frequency of distress (χ^2^ (2) = 4.00, *p* = .14), perceived availability of support (χ^2^ (2) = 5.42, *p* = .07), or perceived quality of support (χ^2^ (2) = 4.79, *p* = .09) across academic, daily living, or socialization areas.

### Social Network Structure

**Fig. 1:**
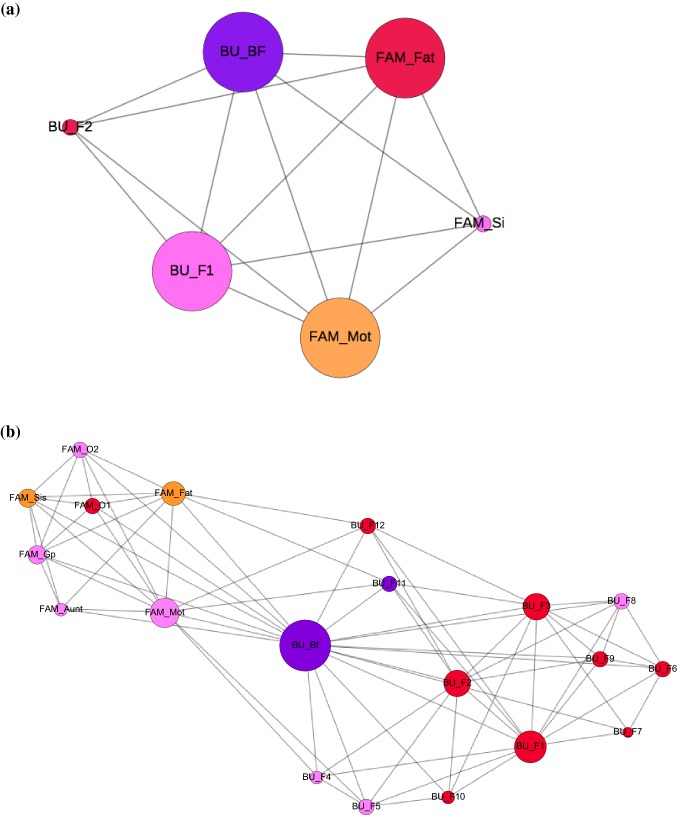

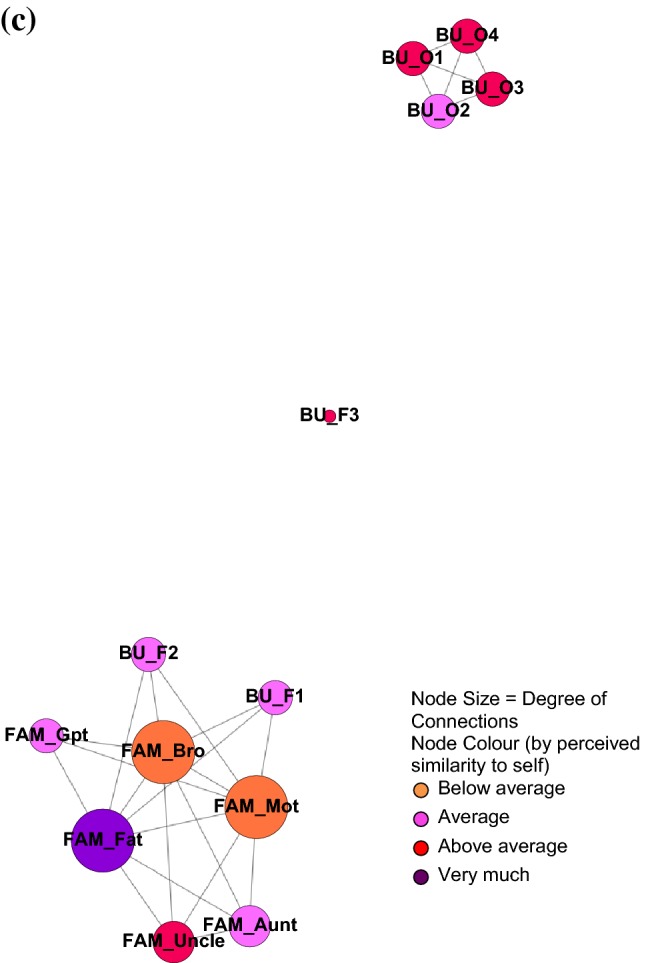
Examples of social network structure of participants from Part 1 feasibility study (N = 10): **a** small network size (5), high network density (0.933); **b** big network size (20), medium network density (0.40); **c** big network size (13), low network density (0.321). *FAM* Family, *Mot* Mother, *Fat* Father, *Gpt* Grandparent, *Sis* Sister, *BU* Before university, *F* Friend, *O* Other, *BF* Boyfriend

Participants reported a wide range of social network sizes and density (Table 2), highlighting a high degree of individual differences in SNS amongst autistic students. Figure 1 illustrates some examples of social networks of various sizes and density. Severity of social communication difficulties was not significantly associated with social network size (r = .13, p = .73), nor social network density (*r* = − .43, *p* = .22). Higher level of social communication difficulties was associated with lower percentage of friends (*r* = − .69, *p* < .05), and higher percentage of other individuals (*r* = .76, *p *< .05) in their social network.

### Perceived Social Support from Social Network

Using Friedman’s test, significant differences were observed for perceived support frequency (Χ^2^ (2) = 12.06, *p* < .01) and quality (Χ^2^ (2) = 8.22, *p* < .05) across different social network members. Post-hoc analyses were conducted using Wilcoxon sign ranked test, with Bonferroni used to correct for multiple comparisons. For perceived frequency of support, participants reported higher frequency of support from family compared to friends (*Z* = − 2.67, *p* < .01), and others (*Z* = − 2.70, *p* < .01), though no differences were found between friends and others (*Z* = − .17, *p* = .87). For perceived quality of support, participants reported higher quality support from family compared to friends (*Z* = − 2.60, *p* < .01), and others (*Z* = − 2.51, *p* < .015), though no differences were found between friends and others (*Z* = − .31, *p* = .75).

## Results Part 2: Convergent Validity of SNaPSS

Participant demographics for Part 2 of the study are shown in Table 3.

**Table 3:** Study Part 2: participant demographics for convergent validity study

	TD (n = 112)		ASD (n = 28)	
	Mean (SD)	Range	Mean (SD)	Range
Age (years)	18.23 (0.46)	17–19	18.32 (0.48)	18–19
AQ-28 total^a^	61.65 (9.48)	36–86	83.07 (10.59)	59–104
SAS-A total^b^	56.82 (12.21)	33–85	70.21 (12.61)	46–89
MSPSS total	5.73 (1.15)	1–7	5.06 (0.99)	3–7
Family	5.77 (1.32)	1–7	5.13 (1.26)	3–7
Friends	5.71 (1.26)	1–7	4.81 (1.33)	1–7
Social network size^c^	11.50 (4.93)	2–20	8.25 (4.83)	0–20
Family (%)	36.14 (17.74)	0–100	36.18 (23.49)	0–80
Friends (%)	58.15 (18.91)	0–100	44.68 (28.72)	0–100
Other (%)	3.09 (6.71)	0–33	9.41 (19.13)	0–81
Social network density^d^	0.35 (0.20)	0–1	0.34 (0.21)	0–0.91
Perceived distress frequency^e^				
Academic	5.29 (3.35)	0–18	8.39 (4.57)	0–16
Daily living	5.72 (3.20)	0–13	8.46 (5.31)	1–18
Social	6.49 (4.86)	0–19	9.53 (6.10)	0–20
Support frequency from network^f^				
Family	6.02 (3.20)	0–13	5.13 (4.30)	0–15
Friends	5.25 (3.32)	0–15	2.93 (3.46)	0–13
Other	0.34 (0.96)	0–5	0.76 (1.66)	0–7
Support quality from network^g^				
Family	9.28 (4.19)	0–15	6.84 (4.94)	0–15
Friends	8.06 (0.43)	0–15	4.68 (5.01)	0–13
Other	0.74 (2.08)	0–10	1.70 (3.55)	0–15

*MSPSS* multidimensional scape of perceived social support

^a^AQ-28 = Autism Quotient-28, is scored between 28 and 112, cut-off is 65

^b^SAS-A = Social anxiety scale for adolescents, scored between 18 and 90, cut-off is 50

^c^Social network size is scored between 0 and 20

^d^Social network density is scored between 0 and 1

^e^Perceived distress frequency is scored between 0 and 20

^f^Support frequency is scored between 0 and 15

^g^Support quality is scored between 0 and 15

### Perceived Distress Frequency

To assess convergent validity between perceived distress frequency and social anxiety amongst TD and autistic students, we conducted Pearson’s correlations between levels of social anxiety (SAS-A total raw score) and perceived distress frequency in academic, daily living, and social areas (Table 4). Higher social anxiety in both TD and autistic students were significantly associated with having higher levels of perceived distress across academic, daily living, and social areas.

**Table 4:** Convergent validity between perceived distress across academic, daily living, and social areas measured by SNaPSS and level of social anxiety, shown by Pearson’s correlation coefficient

	TD			ASD		
	Academic	Daily living	Social	Academic	Daily living	Social
SAS-A total	.366***	.374***	.516***	.412*	.485**	.556**
Academic	–	.396***	.312**	–	.514**	.317
Daily living	–	–	.457***	–	–	.764***
Social	–	–	–	–	–	–

*SNaPSS* social network and perceived social support, *SAS*-*A* social anxiety scale for adolescents

*p < .05, **p < .01, ***p < .001

### Social Network Structure

To assess convergent validity between SNS (SNaPSS) and autistic-like traits amongst TD and autistic students, we conducted Pearson’s correlations between levels of autistic-like traits (AQ-28 total), and participants’ social network size, density, and network composition (Table 5). Amongst TD students, a smaller social network size was associated with having higher levels of autistic-like traits (*r* = − .20, *p* < .05). Neither network density nor composition were significantly associated with levels of autistic-like traits. In contrast, we did not observe any significant correlations between any SNS dimensions and level of autistic-like traits amongst autistic students.

**Table 5:** Convergent validity between social network structure measured by SNaPSS, and level of autistic traits, shown by Pearson’s correlation coefficient

	Size	Density	% FAM	% FRI	% OTH
TD (n = 112)					
AQ-28	− .199*	.034	.137	− .164	.181
Size	–	.302**	− .469***	.464***	.007
Density	–	–	.576***	− .415***	− .174
% FAM	–	–	–	− .906***	− .042
% FRI	–	–	–	–	− .283**
ASD (n = 28)					
AQ-28	.047	− .098	.181	− .229	− .020
Size	–	− .069	− .182	.448*	.010
Density	–	–	.362	− .160	− .029
% FAM	–	–	–	− .401*	− .227
% FRI	–	–	–	–	− .469*

*SNaPSS* social network and perceived social support, *AQ*-*28* Autism quotient-28, *SAS*-*A* social anxiety scale for adolescents, *FAM* Family, *FRI* Friends, *OTH* Other

*p < .05, **p < .01, ***p < .001

### Perceived Social Support

To assess convergent validity between measurements of PSS by SNaPSS and MSPSS (Table 6), we conducted Pearson’s correlations between the perceived frequency and quality of overall support provided by family and friends as measured by SNaPSS, with the family and friends subscale scores of MSPSS. Amongst TD students, we observed good convergent validity, and also adequate discriminant validity. We found that the family subscale from MSPSS only showed significant correlations with the perceived quality and quantity of support from family members as measured by SNaPSS (*r* = .29 to .40, *p* < .01), but not with PSS scores from friends, showing both good convergent and discriminant validity. In contrast, the friends subscale of MSPSS showed significant correlations with the perceived quality and quantity of support from friends as measured by SNaPSS (*r *= .19 to .21, *p* < .05), suggesting good convergent validity. However, the friends’ subscale from MSPSS also showed significant correlation with perceived quality of support from family as measured by SNaPSS (*r *= .23, *p* < .05), suggesting some overlap in PSS from family and friends.

**Table 6:** Convergent validity between perceived social support measured by SNaPSS and MSPSS, shown by Pearson’s correlation coefficient

	MSPSS FRI	FAM Qty	FAM Qlty	FRI Qty	FRI Qlty
TD (n = 112)					
MSPSS FAM	.737***	.288***	.397**	.048	.010
MSPSS FRI	–	.137	.227*	.213*	.190*
FAM Qty	–	–	.800***	.255**	.157
FAM Qlty	–	–	–	.248**	.241**
FRI Qty	–	–	–	–	.834***
ASD (n = 28)					
MSPSS FAM	.108	.230	.287	− .313	− .243
MSPSS FRI	–	.054	.083	.355	.453*
FAM Qty	–	–	.907***	.026	− .057
FAM Qlty	–	–	–	.044	.045
FRI Qty	–	–	–	–	.878***

*SNaPSS* social network and perceived social support, *MSPSS* multidimensional scale of perceived social support, *FAM* Family, *FRI* Friends, *Qty* Quantity, *Qlty* Quality

*p < .05; **p < .01; ***p <.001

Amongst autistic students, we did not observe any significant correlations between the perceived quantity and quality of family support (SNaPSS) and overall family support (MSPSS). For support from friends, we observed significant correlation between the perceived quality of support from friends (SNaPSS) and overall support from friends (MSPSS) (*r* = .45, *p* < .05), though not in the perceived quantity of support.

## Discussion

The current study sought to examine the feasibility and psychometric properties of a novel online tool (the Social Network analysis Perceived Social Support—SNaPSS) designed to measure structural and functional components of social networks among autistic as well as TD students making the transition to university. Firstly, autistic students were able to complete the tool. Their feedback indicated that the tool was clear in its format, use of language, and relevance of items to the different areas related to academic, daily living, and socialization in relation to transition to university, thus indicating the SNaPSS showed good face validity. Based on participants’ verbal and written feedback, two changes are made to the questions included in the questionnaire. First, a question about whether the student has taken a gap year before entering university is added. Second, questions that separately assessed students’ perceived anxiety, stress, and depressed/low mood were combined into a single question that asked about general distress (e.g., anxiety, stress, and depressed/low mood) across academic, daily living, and socialization areas. Combining into a single question will help to reduce both the repetitiveness of the tool, and also help shorten completion time for future research.

Secondly, the tool effectively captured diverse accounts of social networks in terms of structural and functional aspects across this group of autistic students, which can be both quantified and visualized graphically using ecomaps. The graphical representations of networks based on students’ self reports clearly demonstrate the range of complexity and also individual differences in the network composition when broken down by family, friends, and other network members. Using graphical representations to capture changes in social network structure may be especially helpful to summarize and reflect on the dynamic social environment that the student is embedded in at university. Identifying changes in network structure in relation to both size and density may help outline the strengths of social relationships within a social network, and to differentiate between individuals that may be more pivotal or more peripheral in both sustaining connections within the social network structure, and also for providing social support.

Taken together the findings from both SNS and PSS, participants in the feasibility study were able to utilize the novel online tool to help generate an overview of both the structural and functional social network that they perceive to be important to them, and have found the tool to be able to successfully capture a wide range of academic, daily living, and socialization issues during transition to university.

Furthermore, the convergent validity study also gave rise to three key findings. First, both autistic and TD students showed a high positive correlation between social anxiety and perceived distress frequency across academic, daily living, and social domains on the SNaPSS. The breadth of influence that social anxiety had on students’ perceived distress beyond that of the social domain highlights the importance of socialization underlying all aspects of university life. The current findings may not be too surprising in the context of previous research findings, which have shown that students who perceived higher levels of social and emotional support from peers at university experienced better transition outcomes overall at university, and also better mental health (Swenson et al. 2008). Therefore, having the confidence to socialize with others and make new friends at university might not only help alleviate some of the socialization distress, but also help an individual access broader support in academic and daily living areas, thus supporting a better overall transition. It might therefore be helpful for all students, regardless of having autism, to receive some support to recognize, manage, and overcome social anxiety at the start of the academic year, which might in turn help elicit more widespread positive changes in other non-social aspects of the students’ lives.

Second, for SNS, whereas social network size was associated with level of autistic-like traits in TD students, the same pattern was not observed for autistic students. One potential explanation may be that given the AQ offers a broader account of both behavioral and social traits associated with autism, it was more sensitive to detect a broader range of autistic-like traits in the TD student group, and thus the greater variations in AQ scores may have been more sensitive to variations in SNS size. In contrast, the range of AQ scores was much narrower in the small autistic sample, and the smaller individual variance may have reduced statistical power to detect the differences in social network size. Future studies can further evaluate the relationship between autism symptom severity and SNS by using a larger sample of autistic students, and measure autism symptom severity using a variety of clinician, parent, and self-reports to further capture individual variances in autism severity.

Third, the degree of support provided by family and friends as measured by the SNaPSS demonstrated good convergent validity with the MSPSS, another well-validated measure of perceived social support. Some differences were observed in the properties of SNaPSS in this respect between TD and autistic students. There was good convergence between the measures across family and friends’ support as reported by TD students, but only in the friends domain for autistic students. One potential factor that could have caused this discrepancy is that the traditional measures of PSS such as MSPSS place a stronger emphasis on availability of emotional support, rather than more practical aspects of support such as information seeking/daily living (an emphasis of the SNaPSS). Therefore, it may be that for TD students, there is some degree of conflation between reporting the practical and emotional side of PSS from family and friends. TD students may be more likely to turn to the same social contacts both instrumental and emotional support, and instrumental support provided by others may also be perceived to carry some emotional salience. In contrast, this conflation between reporting practical instrumental support and emotional support may be less common amongst autistic students in their self-report. Differences in convergence between SNaPSS and MSPSS for autistic and TD students may therefore be partially due to differences in reporting style. Using a larger sample of autistic students in the future, and focusing on the differences in factual recall of instrumental versus emotional support between autistic and TD students can help further assess factors underlying the differences in convergent validity observed in the current study.

It is important to note that the SNaPSS is developed as a self-report measure, though the use of self-reports in autistic population is often a topic of debate, as some autistic individuals might experience difficulties in introspection as well as emotion recognition, which might influence their ability to consciously report their own experiences (Ben Shalom et al. 2006; Bird et al. 2010; Mazefsky et al. 2011). Some research studies have also found little convergence between autistic individuals’ self- reports of psychological symptoms when compared to parental or clinician report, further challenging whether self-reports are equally valid and accurate in autism research (Mazefsky et al. 2011).

However, one recent systematic review investigating the transition experience of autistic students to university found that the majority of research on recommendations for transition plans and interventions have been theoretically based, with few studies concentrating on the autistic students’ subjective experience of the transition process (Gelbar et al. 2014). The authors highlighted the importance for future research to directly assess the subjective experiences of autistic students at university, and to utilize self-reports of first-hand experiences to better inform evidence-based practice for helping autistic students transitioning to university (Gelbar et al. 2014).

The transitional changes in both SNS and PSS is a subjective and unique experience for each individual, and the current novel online tool SNaPSS therefore provides a structured way for autistic students to report their own personal perception of both their structural and functional social network that they consider to be most important to them. This is especially important as the young person grows older and faces transitional changes such as going to university, as the social changes they are experiencing are unique to that young person, and there may not be a single “other” person who is able to give a holistic perspective as to what the social world of that young person is like across multiple contexts (e.g., home, school, university, work etc.). For example, a family member may only be able to report on how frequently the young person is in contact with family members only, but may be unable to accurately comment on the people that the young person is in contact with at university, or at his/her job. Such limited scope for any single “other” network member to report on the young person’s social world might only provide a very skewed or inaccurate representation of the overall social network of the young person in question. Furthermore, given that the SNaPSS focuses on the young person’s *perception* of their personal SNS and PSS, it may be difficult for others to accurately report on what they believe to be what the young person perceives their social world to be, and which people the young person considers to be closest to him/her. Although an other-user version of SNaPSS may enable a specific network member perceived to be closed to the younger person to provide validation for a single domain of an individual’s social network and perception of support provided by that domain only (such as family, school, university, or work etc.), this would be of limited utility in respect of the measurement tool as a whole to capture an individual’s holistic social world. Therefore, although the use of self-report may suffer from reporting bias as a limitation, in the case of transitioning to university or adulthood, the nature of using self-report for the purpose of SNaPSS is both necessary and essential.

Providing insight into autistic students’ perception of support from various social network members can be particularly informative for university stakeholders to adopt a more holistic and systemic perspective when formulating transition plans. The current tool (SNaPSS) can therefore help stakeholders monitor how best to integrate different social resources such as family, peers, and university staff to ensure both a continuation of support during transition to university, and that each type of social network member can provide more specialized and efficient support to meet students’ needs.

## Limitations

It is also important to consider some limitations of the current study. Although the current study found good feasibility and face validity of the SNaPSS as a novel tool to measure SNS and PSS amongst autistic students, convergent validity for PSS and SNS is somewhat inconsistent across TD and autistic students. Future research can use a larger sample of autistic students to further assess whether there are differences in reporting emotional and instrumental PSS between autistic and TD students, as well as conducting further analysis into the types of social communication deficits experienced by autistic students that have the most significant impact on their SNS when compared to TD students.

Furthermore, the current feasibility and convergent validity studies are both cross-sectional and only used a small sample (n = 10, 28 respectively) of autistic students either prior to, or at the start of their transition to university. This is a limitation for two main reasons. First, it is unclear whether the SNaPSS may be sensitive to detect changes in SNS and PSS over time, as students transition through their first year of university life. More longitudinal design that use the SNaPSS to monitor student transition over time may help assess SNaPSS’s sensitivity to change, which is important as a tool that assesses the dynamic social network and perceived social support structure. Second, despite the clear diagnostic criteria set out for autism in the DSM-5, it is a highly heterogeneous condition, especially in terms of sex-related differences in behavioral presentation (Dean et al. 2017), as well as many co-occurring mental and physical health issues experienced by many autistic individuals. Therefore, the generalizability of current findings based on a small sample of autistic students is rather limited, and future studies should seek to replicate current findings using a larger and more diverse sample of autistic students at university.

## Future Research

Future studies should seek to adopt a longitudinal design over the first year of university life to help monitor *changes* in both SNS and PSS during the transition process. This would better assess whether changes in either SNS and/or PSS observed may be associated with university transition outcomes in either student group. Characterizing differences in how changes in SNS and PSS can influence transition outcomes can help university stakeholders design more tailored interventions to better support each student group during their university transition, further enhancing students’ university experience.

The current SNaPSS is a novel online tool that uses ecomap structure to capture both quantitatively and qualitatively the unique SNS and PSS from network members that an individual perceives to be close to them. The current format of SNaPSS focuses on students’ social relationships throughout the transition to traditional college attendance, the measure could also be adapted for autistic students who are making the transition to post-secondary education delivered via distance learning or online attendance. This would involve asking students to consider online and offline social contacts separately when completing the social network structure section of the SNaPSS, participant can then explicitly state which social network members they have concluded are online only, offline only, or both. This would enable the construction of different types of ecomaps depending on the researcher’s interest as to whether to investigate the combined social network structure, or one mode only.

Next, although the current SNaPSS tool focuses on using self-report tool to highlight an individual’s perception of their *personal* social world, one potential future direction is to evaluate whether the development of an other-user version of SNaPSS may be more useful for use with younger population (such as autistic school-age children) or with individuals with intellectual disability, who might be unable to accurately generate self-report of their overall social network, and need to rely on adults who are working closely with them to help report their social network structure and perceived social support.

Beyond the focus for examining areas associated with transition to university per se, the tool can be adapted for use with other populations, and examine other areas of support both during other important life transitions, such as school, employment, and aging, but also may be helpful as a way to routinely monitor an individual’s closest social world. The structure and format of questions included in the SNaPSS can serve as a framework for measuring SNS and PSS more broadly, and future research can adapt the tool for use beyond the current university student population, and assess the broader face validity of the SNaPSS across multiple settings.

## Footnotes

1 In a recently published article (Kenny et al. 2016), members of the autism community preferred to use identity-first language (i.e., autistic individual), whereas professionals preferred to use person-first language (i.e., individual with autism). Here we choose to use identity-first language, although we are aware that some might prefer to use person-first language.

2 In the UK, a gap year is an optional year out of education between finishing secondary education, and starting post-secondary education.
